# Comparative Effectiveness and Antibody Responses to Moderna and Pfizer-BioNTech COVID-19 Vaccines among Hospitalized Veterans — Five Veterans Affairs Medical Centers, United States, February 1–September 30, 2021

**DOI:** 10.15585/mmwr.mm7049a2

**Published:** 2021-12-10

**Authors:** Kristina L. Bajema, Rebecca M. Dahl, Steve L. Evener, Mila M. Prill, Maria C. Rodriguez-Barradas, Vincent C. Marconi, David O. Beenhouwer, Mark Holodniy, Cynthia Lucero-Obusan, Sheldon T. Brown, Maraia Tremarelli, Monica Epperson, Lisa Mills, So Hee Park, Gilberto Rivera-Dominguez, Rosalba Gomez Morones, Ghazal Ahmadi-Izadi, Rijalda Deovic, Chad Mendoza, Chan Jeong, Stephanie J. Schrag, Elissa Meites, Aron J. Hall, Miwako Kobayashi, Meredith McMorrow, Jennifer R. Verani, Natalie J. Thornburg, Diya Surie, SUPERNOVA COVID-19, Surveillance Group, Surveillance Platform for Enteric and Respiratory Infectious Organisms at the VA (SUPERNOVA) COVID-19 Surveillance Group, Joy Burnette, Gustavo Capo, Lauren Epstein, Julia Gallini, Telisha Harrison, Amy Hartley, Liliana Hernandez, Elena Morales, Nina Patel, Kim Rooney, Tehquin Tanner, Ernest Tate, Ashley Tunson, Alexis Whitmire, Juton Winston, Katherine Elliot, Ilda Graham, Diki Lama, Ismael Pena, Adrienne Perea, Guerry Anabelle Perez, Johane Simelane, Sarah Smith, Gabriela Tallin, Amelia Tisi, Alonso Arellano Lopez, Miguel Covarrubias Gonzalez, Bashir Lengi, Mariana Vanoye Tamez, Babak Aryanfar, Ian Lee-Chang, Anthony Matolek, Aleksandra Poteshkina, Saadia Naeem, Evan Goldin, Madhuri Agrawal, Jessica Lopez, Theresa Peters, Geliya Kudryavtseva, Jordan Cates, Anita Kambhampati

**Affiliations:** ^1^CDC COVID-19 Response Team; ^2^Karna, LLC, Atlanta, Georgia; ^3^Michael E. DeBakey Veterans Affairs Medical Center, Houston, Texas; ^4^Department of Medicine, Baylor College of Medicine, Houston, Texas; ^5^Atlanta VA Medical Center, Atlanta, Georgia; ^6^Department of Medicine, Emory University School of Medicine, Atlanta, Georgia; ^7^Department of Global Health, Rollins School of Public Health, Emory University, Atlanta, Georgia; ^8^Veterans Affairs Greater Los Angeles Healthcare System, Los Angeles, California; ^9^Department of Medicine, David Geffen School of Medicine at UCLA, Los Angeles, California; ^10^Veterans Affairs Palo Alto Health Care System, Palo Alto, California; ^11^Public Health Program Office, Department of Veterans Affairs, Washington, DC; ^12^Department of Medicine, Stanford University, Stanford, California; ^13^Department of Medicine, Icahn School of Medicine at Mount Sinai, New York, New York; ^14^James J. Peters Veterans Affairs Medical Center, Bronx, New York, New York; ^15^General Dynamics Information Technology, Falls Church, Virginia.; Atlanta Veterans Affairs Medical Center, Atlanta, Georgia; Atlanta Veterans Affairs Medical Center, Atlanta, Georgia; Atlanta Veterans Affairs Medical Center, Atlanta, Georgia; Atlanta Veterans Affairs Medical Center, Atlanta, Georgia; Atlanta Veterans Affairs Medical Center, Atlanta, Georgia; Atlanta Veterans Affairs Medical Center, Atlanta, Georgia; Atlanta Veterans Affairs Medical Center, Atlanta, Georgia; Atlanta Veterans Affairs Medical Center, Atlanta, Georgia; Atlanta Veterans Affairs Medical Center, Atlanta, Georgia; Atlanta Veterans Affairs Medical Center, Atlanta, Georgia; Atlanta Veterans Affairs Medical Center, Atlanta, Georgia; Atlanta Veterans Affairs Medical Center, Atlanta, Georgia; Atlanta Veterans Affairs Medical Center, Atlanta, Georgia; Atlanta Veterans Affairs Medical Center, Atlanta, Georgia; Atlanta Veterans Affairs Medical Center, Atlanta, Georgia; James J. Peters Veterans Affairs Medical Center, Bronx, New York; James J. Peters Veterans Affairs Medical Center, Bronx, New York; James J. Peters Veterans Affairs Medical Center, Bronx, New York; James J. Peters Veterans Affairs Medical Center, Bronx, New York; James J. Peters Veterans Affairs Medical Center, Bronx, New York; James J. Peters Veterans Affairs Medical Center, Bronx, New York; James J. Peters Veterans Affairs Medical Center, Bronx, New York; James J. Peters Veterans Affairs Medical Center, Bronx, New York; James J. Peters Veterans Affairs Medical Center, Bronx, New York; James J. Peters Veterans Affairs Medical Center, Bronx, New York; Michael E. DeBakey Veterans Affairs Medical Center, Houston, Texas; Michael E. DeBakey Veterans Affairs Medical Center, Houston, Texas; Michael E. DeBakey Veterans Affairs Medical Center, Houston, Texas; Michael E. DeBakey Veterans Affairs Medical Center, Houston, Texas; Veterans Affairs Greater Los Angeles Healthcare System, Los Angeles, California; Veterans Affairs Greater Los Angeles Healthcare System, Los Angeles, California; Veterans Affairs Greater Los Angeles Healthcare System, Los Angeles, California; Veterans Affairs Greater Los Angeles Healthcare System, Los Angeles, California; Veterans Affairs Greater Los Angeles Healthcare System, Los Angeles, California; Veterans Affairs Greater Los Angeles Healthcare System, Los Angeles, California; Veterans Affairs Palo Alto Health Care System, Palo Alto, California; Veterans Affairs Palo Alto Health Care System, Palo Alto, California; Veterans Affairs Palo Alto Health Care System, Palo Alto, California; Veterans Affairs Palo Alto Health Care System, Palo Alto, California; CDC; CDC

The mRNA COVID-19 vaccines (Moderna and Pfizer-BioNTech) provide strong protection against severe COVID-19, including hospitalization, for at least several months after receipt of the second dose (*1*,*2*). However, studies examining immune responses and differences in protection against COVID-19–associated hospitalization in real-world settings, including by vaccine product, are limited. To understand how vaccine effectiveness (VE) might change with time, CDC and collaborators assessed the comparative effectiveness of Moderna and Pfizer-BioNTech vaccines in preventing COVID-19–associated hospitalization at two periods (14–119 days and ≥120 days) after receipt of the second vaccine dose among 1,896 U.S. veterans at five Veterans Affairs medical centers (VAMCs) during February 1–September 30, 2021. Among 234 U.S. veterans fully vaccinated with an mRNA COVID-19 vaccine and without evidence of current or prior SARS-CoV-2 infection, serum antibody levels (anti-spike immunoglobulin G [IgG] and anti-receptor binding domain [RBD] IgG) to SARS-CoV-2 were also compared. Adjusted VE 14–119 days following second Moderna vaccine dose was 89.6% (95% CI = 80.1%–94.5%) and after the second Pfizer-BioNTech dose was 86.0% (95% CI = 77.6%–91.3%); at ≥120 days VE was 86.1% (95% CI = 77.7%–91.3%) for Moderna and 75.1% (95% CI = 64.6%–82.4%) for Pfizer-BioNTech. Antibody levels were significantly higher among Moderna recipients than Pfizer-BioNTech recipients across all age groups and periods since vaccination; however, antibody levels among recipients of both products declined between 14–119 days and ≥120 days. These findings from a cohort of older, hospitalized veterans with high prevalences of underlying conditions suggest the importance of booster doses to help maintain long-term protection against severe COVID-19.^†^

During February 1–September 30, 2021, adults aged ≥18 years hospitalized at five VAMCs (Atlanta, Georgia; the New York City borough of the Bronx; Houston, Texas; Los Angeles, California; and Palo Alto, California) were screened for inclusion in this test-negative case-control assessment (*1*,*3*). Patients with COVID-19–like illness^§^ who received a positive SARS-CoV-2 nucleic acid amplification test result were included as case-patients and those with COVID-19–like illness and negative SARS-CoV-2 test results were included as controls^¶^ (*4*).

Data on demographic characteristics, clinical history, and COVID-19 vaccination history were abstracted from electronic health records.** Full vaccination was defined as receipt of 2 doses of an mRNA COVID-19 vaccine (Moderna or Pfizer-BioNTech) ≥14 days before the SARS-CoV-2 test. Participants who received only 1 dose of an mRNA COVID-19 vaccine, 2 mRNA doses with receipt of the second dose <14 days before the SARS-CoV-2 test, mixed mRNA vaccine products, 3 vaccine doses, or the Janssen (Johnson & Johnson) COVID-19 vaccine were excluded from the analysis.^††^

Available residual clinical serum specimens were collected from fully vaccinated hospitalized control patients at all sites and tested at CDC. Specimens were tested using the V-PLEX SARS-CoV-2 panel 2 kit (Meso Scale Diagnostics)^§§^ to measure binding IgG levels against three SARS-CoV-2 antigens: the spike protein (anti-spike), the receptor-binding domain of the spike protein (anti-RBD), and the nucleocapsid protein (anti-nucleocapsid). Levels were reported in international binding antibody units (BAU) per milliliter (mL). Control participants with anti-nucleocapsid antibodies (>11.8 BAU/mL), suggesting a prior SARS-CoV-2 infection, were excluded from the final analysis.

VE to prevent COVID-19–associated hospitalization (calculated as 1 – adjusted odds ratio [aOR] × 100)^¶¶^ was estimated using multivariable logistic regression to compare the odds of full vaccination between case-patients and controls. Models were adjusted for VAMC site, admission date, and age (with the use of cubic splines), sex, and race/ethnicity.*** VE between subgroups was compared using 95% CIs. In the antibody analysis, pairwise comparisons of median anti-spike IgG and anti-RBD IgG levels using the Wilcoxon rank-sum test and p-values were calculated among participants by age category, vaccine product received, and time since vaccination (14–119 days and ≥120 days after the second vaccine dose). Because vaccines might not elicit a strong immune response^†††^ in some persons with immunocompromising conditions,^§§§^ differences including and excluding this group were examined. Analyses were conducted using SAS (version 9.4; SAS Institute). For all analyses, statistical significance was set at p<0.05. Protocols were reviewed and approved by the VAMC Research and Development Committee at each site. The activity was also reviewed by CDC and conducted consistent with applicable federal law and CDC policy.^¶¶¶^

During February 1–September 30, 2021, a total of 2,329 hospitalized U.S. veterans with COVID-19–like illness met inclusion criteria. After excluding 433 persons with missing data or ineligible vaccination status,**** 755 case-patients and 1,141 controls were included in the analysis. Among these 1,896 patients, 1,758 (92.7%) were male, the median age was 67 years (IQR = 59–75 years), 942 (49.7%) were Black, and 162 (8.5%) were Hispanic (Table 1). Effectiveness of the Moderna vaccine was 89.6% (95% CI = 80.1%–94.5%) 14–119 days after the second vaccine dose and 86.1% (95% CI = 77.7%–91.3%) at ≥120 days (Table 2). Effectiveness of the Pfizer-BioNTech vaccine was 86.0% (95% CI = 77.6%–91.3%) at 14–119 days and 75.1% (95% CI = 64.6%–82.4%) at ≥120 days.

**TABLE 1:** Characteristics of COVID-19 case-patients and controls* among hospitalized veterans — five Veterans Affairs medical centers, United States, February 1–September 30, 2021

Characteristic	No. (%)		
	Total N = 1,896	Case-patients n = 755	Controls n = 1,141
**Male sex**	**1,758 (92.7)**	**679 (89.9)**	**1,079 (94.6)**
**Age, median (IQR), yrs**	67 (59–75)	63 (51–74)	70 (62–76)
**Age group, yrs**			
18–49	241 (12.7)	166 (22.0)	75 (6.6)
50–64	551 (29.1)	238 (31.5)	313 (27.4)
65–74	621 (32.8)	189 (25.0)	432 (37.9)
75–84	334 (17.6)	114 (15.1)	220 (19.3)
≥85	149 (7.9)	48 (6.4)	101 (8.9)
**Race/Ethnicity**			
Black, non-Hispanic	942 (49.7)	377 (49.9)	565 (49.5)
White, non-Hispanic	748 (39.5)	277 (36.7)	471 (41.3)
Hispanic, any race	162 (8.5)	82 (10.9)	80 (7.0)
Other, non-Hispanic^†^	44 (2.3)	19 (2.5)	25 (2.2)
**Resident in long-term care facility^§^ (unknown = 20)**	114 (6.1)	28 (3.7)	86 (7.6)
**Study site**			
Atlanta, Georgia	615 (32.4)	243 (32.2)	372 (32.6)
Bronx, New York City**^¶^**	102 (5.4)	33 (4.4)	69 (6.0)
Houston, Texas	713 (37.6)	372 (49.3)	341 (29.9)
Los Angeles, California	328 (17.3)	74 (9.8)	254 (22.3)
Palo Alto, California	138 (7.3)	33 (4.4)	105 (9.2)
**Month of admission**			
Feb–Mar	451 (23.8)	151 (20.0)	300 (26.3)
Apr–Jun	442 (23.3)	118 (15.6)	324 (28.4)
Jul–Sep	1,003 (52.9)	486 (64.4)	517 (45.3)
COVID-19 fully vaccinated**	799 (42.1)	161 (21.3)	638 (55.9)
**COVID-19 vaccine type among fully vaccinated**			
Pfizer BioNTech	521 (65.2)	118 (73.3)	403 (63.2)
Moderna	278 (34.8)	43 (26.7)	235 (36.8)
**Time between vaccine dose 2 and SARS-CoV-2 test among fully vaccinated, median (IQR), days**	130 (70–169)	157 (125–184)	120 (63–163)
**Underlying medical condition**			
**Cardiovascular**			
Atherosclerotic cardiovascular disease^††^	538 (29.2)	157 (22.0)	381 (33.8)
Atrial fibrillation	265 (14.0)	88 (11.7)	177 (15.5)
Congestive heart failure	428 (22.6)	94 (12.5)	334 (29.3)
Hypertension	1,312 (69.2)	478 (63.3)	834 (73.1)
Venous thromboembolism	110 (5.8)	41 (5.4)	69 (6.0)
**Metabolic**			
Diabetes	805 (42.5)	300 (39.7)	505 (44.3)
Dyslipidemia	813 (42.9)	296 (39.2)	517 (45.3)
Obesity^§§^ (unknown = 3)	897 (47.4)	396 (52.6)	501 (43.9)
**Pulmonary**			
Asthma	125 (6.6)	36 (4.8)	89 (7.8)
COPD or emphysema	442 (23.3)	94 (12.5)	348 (30.5)
Obstructive sleep apnea	352 (18.6)	142 (18.8)	210 (18.4)
**Neurologic**			
Dementia	111 (5.9)	39 (5.2)	72 (6.3)
Stroke or transient ischemic attack	188 (9.9)	60 (7.9)	128 (11.2)
**Renal**			
Chronic kidney disease	372 (19.6)	122 (16.2)	250 (21.9)
End stage renal disease, on dialysis	82 (4.3)	19 (2.5)	63 (5.5)
**Liver**			
Liver disease	165 (8.7)	50 (6.6)	115 (10.1)
**Immunocompromising condition**			
Immunocompromise or therapy**^¶¶^**	275 (14.9)	64 (9.0)	211 (18.8)
Tobacco use***			
Current	347 (18.3)	91 (12.1)	256 (22.4)
Former	559 (29.5)	170 (22.5)	389 (34.1)
**No. of hospitalizations during past year (unknown = 45)**			
0	1,138 (61.5)	534 (72.7)	604 (54.1)
1	364 (19.7)	120 (16.3)	244 (21.9)
≥2	349 (18.9)	81 (11.0)	268 (24.0)
**Outcome**			
Intensive care unit admission (unknown = 10)	392 (20.7)	179 (23.8)	213 (18.7)
Death (unknown = 12)	108 (5.7)	64 (8.6)	44 (3.9)

**Abbreviations:** COPD = chronic obstructive pulmonary disease; VAMC = Veterans Affairs medical center.

* Case-patients were defined as patients with COVID-19–like illness (i.e., presence of fever, new or worsened cough or shortness of breath, loss of taste or smell, oxygen saturation on room air <94%, requirement for noninvasive ventilation or endotracheal intubation with mechanical ventilation, or chest radiograph or computed tomography pulmonary findings consistent with pneumonia) who tested positive for SARS-CoV-2 by nucleic acid amplification test performed within 14 days before admission or during the first 72 hours of hospitalization. Controls were defined as patients with COVID-19–like illness and negative SARS-CoV-2 test results during the same period.

^†^ Included non-Hispanic American Indian and Alaska Native, non-Hispanic Asian and Other Pacific Islander, non-Hispanic multiple races, or non-Hispanic Other race.

^§^ Included residence before admission to VAMC and non-VAMC nursing facilities as well as other VAMC long-term housing (e.g., domiciliary).

**^¶^** The Bronx is a borough in New York City.

** COVID-19 vaccination status includes unvaccinated, defined as no receipt of any SARS-CoV-2 vaccine, and fully vaccinated, defined as receipt of both doses of an mRNA (Pfizer-BioNTech or Moderna) ≥14 days before the first SARS-CoV-2 test performed within 14 days before admission or during the first 72 hours of hospitalization.

^††^ Included coronary artery disease, myocardial infarction, peripheral vascular disease, carotid artery stenosis.

^§§^ Body mass index ≥30 kg/m^2^.

**^¶¶^** Included HIV/AIDS, malignancy, history of solid organ or stem cell transplant, or immunosuppressive therapy (systemic steroids, chemotherapy, or other immunosuppressive therapy within 1 month of SARS-CoV-2 test).

*** Tobacco use was defined as smoking of cigarettes, cigars, or pipes. Current use of tobacco was defined as use within the previous 12 months of hospitalization, whereas former use occurred >12 months before hospitalization.

**TABLE 2:** Characteristics of case-patients and controls and adjusted effectiveness* of full vaccination^†^ with mRNA COVID-19 vaccines against COVID-19–associated hospitalization among veterans — five Veterans Affairs medical centers,^§^ United States, February 1–September 30, 2021

Characteristic	No./Total no. (%)		Adjusted VE % (95% CI)
	Case-patients vaccinated/total	Controls vaccinated/total	
**Overall**	**161/755 (21.3)**	**638/1,141 (55.9)**	**83.7 (78.8–87.5)**
**Age group, yrs**			
**18–64**			
Pfizer-BioNTech and Moderna vaccine products	33/404 (8.2)	164/388 (42.3)	92.2 (87.4–95.2)
Pfizer-BioNTech	23/404 (5.7)	86/388 (22.2)	89.4 (80.9–94.1)
Moderna	10/404 (2.5)	78/388 (20.1)	94.5 (88.4–97.4)
**≥65**			
Pfizer-BioNTech and Moderna vaccine products	128/351 (36.5)	474/753 (62.9)	75.6 (66.2–82.4)
Pfizer-BioNTech	95/351 (27.1)	317/753 (42.1)	72.9 (61.1–81.2)
Moderna	33/351 (9.4)	157/753 (20.8)	78.6 (64.9–86.9)
**COVID-19 vaccine product^†^**			
**Pfizer-BioNTech**			
All periods since vaccination**^¶^**	118/755 (15.6)	403/1,141 (35.3)	79.8 (72.7–85.1)
14–119 days	26/755 (3.4)	200/1,141 (17.5)	86.0 (77.6–91.3)
≥120 days	92/755 (12.2)	203/1,141 (17.8)	75.1 (64.6–82.4)
**Moderna**			
All periods since vaccination**^¶^**	43/755 (5.7)	235/1,141 (20.6)	87.0 (80.7–91.2)
14–119 days	12/755 (1.6)	119/1,141 (10.4)	89.6 (80.1–94.5)
≥120 days	31/755 (4.1)	116/1,141 (10.2)	86.1 (77.7–91.3)
**No. of days since vaccination, age group**			
**14–119 days**			
≥18 yrs	38/755 (5.0)	319/1,141 (28.0)	87.8 (81.8–91.7)
18–64 yrs	8/404 (2.0)	89/388 (22.9)	95.1 (89.1–97.8)
≥65 yrs	30/351 (8.5)	230/753 (30.5)	81.2 (69.9–88.2)
**≥120 days**			
≥18 yrs	123/755 (16.3)	319/1,141 (28.0)	80.0 (72.7–85.4)
18–64 yrs	25/404 (6.2)	75/388 (19.3)	89.2 (80.8–93.9)
≥65 yrs	98/351 (27.9)	237/753 (31.5)	72.9 (60.0–81.7)

**Abbreviation:** VE = vaccine effectiveness.

*All nonstratified models adjusted for study site, time (admission date), age, sex, and race/ethnicity. Stratified models exclude adjustment for stratification variable.

^†^ Includes unvaccinated, defined as no receipt of any SARS-CoV-2 vaccine, and fully vaccinated, defined as receipt of both doses of an mRNA (Pfizer-BioNTech or Moderna) ≥14 days before the first SARS-CoV-2 test performed within 14 days before admission or during the first 72 hours of hospitalization.

^§^ The five Veterans Affairs medical centers are located in Atlanta, Georgia; the New York City borough of the Bronx; Houston, Texas; Los Angeles, California; and Palo Alto, California.

**^¶^** Among fully vaccinated, time since second dose of COVID-19 mRNA vaccine.

Antibody testing was performed on sera available from 259 of 638 (40.6%) fully vaccinated controls. No significant differences in age, sex, or vaccine product received were observed between fully vaccinated controls with and without available sera (Supplementary Table 1, https://stacks.cdc.gov/view/cdc/112103). After excluding 25 (9.7%) control specimens with anti-nucleocapsid antibodies, the analysis included 90 (38.5%) controls fully vaccinated with the Moderna vaccine (median age = 72 years; median interval from second dose to serum collection = 75 days; 24 [26.7%] with an immunocompromising condition) and 144 (61.5%) who were fully vaccinated with the Pfizer-BioNTech vaccine (median age = 73 years; median interval from second dose to serum collection = 102 days; 38 [26.4%] with an immunocompromising condition). Among fully vaccinated Moderna controls, anti-spike IgG levels were higher among persons with sera collected 14–119 days after the second vaccine dose (median = 759 BAU/mL; IQR = 348–2,086 BAU/mL) compared with ≥120 days (median = 266 BAU/mL; IQR = 133–441 BAU/mL) (p = 0.002) (Figure). Anti-spike IgG levels were also higher among fully vaccinated Pfizer-BioNTech controls at 14–119 days after receipt of dose 2 (median = 187 BAU/mL; IQR = 50–493 BAU/mL) than at ≥120 days (median = 62 BAU/mL; IQR = 25–141 BAU/mL) (p = 0.001). At 14–119 days after the second dose, anti-spike IgG levels were higher among controls fully vaccinated with the Moderna vaccine compared with those who received the Pfizer-BioNTech vaccine among persons aged 18–64 years (median = 612 versus 340; p = 0.018) and ≥65 years (median = 792 versus 152; p<0.001). At ≥120 days, anti-spike IgG levels were also higher among controls fully vaccinated with the Moderna vaccine compared with the Pfizer-BioNTech vaccine among persons aged 18–64 years (median = 267 versus 106; p = 0.006) and ≥65 years (median = 266 versus 57; p = 0.003). Relative differences in anti-RBD IgG levels across groups were similar to differences in anti-spike IgG levels (Supplementary Table 2, https://stacks.cdc.gov/view/cdc/112104), and differences in anti-SARS-CoV-2 antibody levels were similar across groups with immunocompromised persons included or excluded from the analysis.

**FIGURE:**
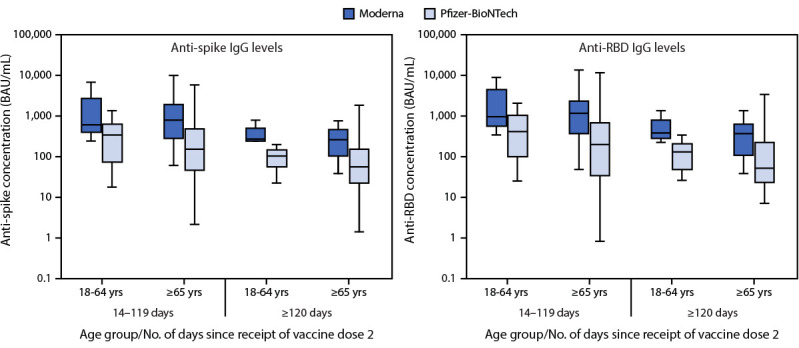
Serum anti-spike and anti-receptor binding domain immunoglobulin G levels* after full vaccination among hospitalized veterans without current or previous SARS-CoV-2 infection^†^ — five Veterans Affairs medical centers,^§^ United States, February 1–September 30, 2021^⁋^ **Abbreviations:** BAU = binding antibody units; IgG = immunoglobulin G; RBD = receptor binding domain. * Anti-spike and anti-RBD IgG levels were measured in sera of hospitalized veterans collected at or within 2 days of hospital admission. In these box and whisker plots, the central horizontal line of each box plot represents the median, with the box denoting the IQR and the whiskers representing 1.5 x IQR. ^†^ Excluded 25 controls with anti-nucleocapsid antibodies (>11.8 BAU/mL), suggesting a previous SARS-CoV-2 infection. ^§^ The five Veterans Affairs medical centers are located in Atlanta, Georgia; the New York City borough of the Bronx; Houston, Texas; Los Angeles, California; and Palo Alto, California. ^¶^ Serum specimens collected during March 22**–**August 31, 2021.

## Discussion

Among U.S. veterans hospitalized at five VAMCs during February–September 2021, mRNA COVID-19 vaccines remained effective in preventing COVID-19–associated hospitalizations ≥120 days after receipt of the second dose of Moderna (VE = 86%) or Pfizer-BioNTech vaccines (VE = 75%). Among recipients of Moderna and Pfizer-BioNTech vaccines, anti-SARS-CoV-2 spike and RBD IgG levels declined with increasing time since vaccination, although U.S. veterans who received the Moderna vaccine consistently had higher antibody levels compared with recipients of the Pfizer-BioNTech vaccine across age groups and time since vaccination. These findings from a cohort of older, hospitalized veterans with high prevalences of underlying conditions suggest the importance of booster doses to help maintain long-term protection against severe COVID-19. 

 Although an immune correlate of protection for COVID-19 vaccination has yet to be established, studies have shown a relationship between binding antibody levels, neutralizing antibody levels, and vaccine efficacy in clinical trials (*5*, *6*). Pairing antibody levels from the same population in which COVID-19 VE is estimated can inform how changes in humoral immunity relate to real-world protection against COVID-19. Although this analysis was not powered to detect small differences in VE by mRNA product as seen in other hospitalized settings (*7*), significantly higher post-Moderna vaccination antibody levels compared with Pfizer-BioNTech were observed, which is consistent with findings from other studies (*7*,*8*). Potential reasons for this difference include higher antigen content and a longer interval between doses for the Moderna vaccine compared with the Pfizer-BioNTech vaccine (*8*). Overall, for both vaccine products, antibody levels in this cohort of older U.S. veterans with high prevalences of underlying medical conditions were substantially lower than levels seen among younger, healthy volunteers or health care personnel in other studies (*7*,*9*). Consistent with results from studies among younger, healthy persons, antibody levels appeared to wane over time but remained detectable ≥120 days after vaccination (*9*,*10*). Although not statistically significant, VE point estimates also declined between 14–119 days and ≥120 days from receipt of second vaccine dose.

The findings in this report are subject to at least four limitations. First, there was insufficient statistical power to detect potential small differences in VE by vaccine product or period since vaccination. Second, it was not possible to assess antibody levels or VE beyond 4 months since receipt of second vaccine dose. Third, residual clinical sera were only available from 41% of fully vaccinated controls. Finally, binding antibody levels are a surrogate correlate of protection against SARS-CoV-2 and other components of immunity, such as cell-mediated immune responses, were not measured.

Both mRNA COVID-19 vaccines that are approved by the Food and Drug Administration or authorized for use in the United States remain effective against COVID-19–associated hospitalization among U.S. veterans. Antibody levels in this cohort of older persons with high prevalences of underlying medical conditions were lower than those in younger, healthier populations and declined over time. Continued monitoring of the effectiveness of COVID-19 vaccines alongside anti-SARS-CoV-2 antibody levels is needed to better understand the duration of protection of these vaccines and the correlation of antibody levels with protection. These findings suggest the importance of booster doses to help maintain long-term protection against severe COVID-19.

**Summary**

**What is already known about this topic?**

mRNA COVID-19 vaccines are effective in preventing severe COVID-19. Some studies have shown declines in vaccine effectiveness against severe COVID-19 with increasing time since vaccination.

**What is added by this report?**

During February 1–September 30, 2021, mRNA vaccine effectiveness in preventing COVID-19–associated hospitalizations among U.S. veterans ≥120 days after receipt of the second dose was 86% for Moderna and 75% for Pfizer-BioNTech vaccines. Antibody responses to both vaccines decreased over time. Moderna vaccine recipients had higher antibody levels than did Pfizer-BioNTech recipients.

**What are the implications for public health practice?**

These findings from a cohort of older, hospitalized veterans with high prevalences of underlying conditions suggest the importance of booster doses to help maintain long-term protection against severe COVID-19.

## Footnotes

* These authors contributed equally to this report.

†
https://www.cdc.gov/coronavirus/2019-ncov/vaccines/booster-shot.html

§ COVID-19–like illness was defined as fever, new or worsened cough or shortness of breath, loss of taste or smell, oxygen saturation on room air <94%, requirement for noninvasive ventilation or endotracheal intubation with mechanical ventilation, or chest radiograph or computed tomography pulmonary findings consistent with pneumonia.

¶ The test-negative study design is commonly used to assess vaccine effectiveness in observational studies. In this study design, case-patients with symptomatic COVID-19 who test positive for SARS-CoV-2 are compared with controls with the same clinical syndrome who test negative for SARS-CoV-2. This approach is used to reduce bias from differences in health care-seeking behavior and access to testing and care.

** In the Atlanta and Houston VAMCs, COVID-19 vaccination status was further verified through a review of state immunization registries.

†† Sixty-one participants received the Janssen (Johnson & Johnson) COVID-19 vaccine and were therefore excluded from the analysis.

§§
https://www.mesoscale.com/en/products/sars-cov-2-panel-2-igg-k15383u/

¶¶
https://www.who.int/publications/i/item/WHO-2019-nCoV-vaccine_effectiveness-measurement-2021.1

*** Additional factors were included if they changed the aOR by ≥5% when added individually to the base model.

†††
https://www.cdc.gov/coronavirus/2019-ncov/science/science-briefs/fully-vaccinated-people.html

§§§ Included HIV/AIDS, malignancy, history of solid organ or stem cell transplant, or receipt of immunosuppressive therapy (systemic steroids, chemotherapy, or other immunosuppressive therapy) within 1 month of SARS-CoV-2 test.

¶¶¶ 45 C.F.R. part 46.102(l)(2), 21 C.F.R. part 56; 42 U.S.C. Sect. 241(d); 5 U.S.C. Sect. 552a; 44 U.S.C. Sect. 3501 et seq.

**** Included 103 veterans with missing demographic data, vaccination date, or product information; 260 who received only 1 dose of mRNA COVID-19 vaccine or 2 doses <14 days before the qualifying SARS-CoV-2 test; three who received mixed mRNA COVID-19 vaccine products; 61 who received the Janssen (Johnson & Johnson) COVID-19 vaccine; and six fully vaccinated persons who received a third vaccine dose.
